# Over-Referred and Badly Served – Patient Journeys in Attention Deficit Hyperactivity Disorder at a Community Mental Health Team

**DOI:** 10.1192/bjo.2024.464

**Published:** 2024-08-01

**Authors:** Samuel Brooks, Delia Annear

**Affiliations:** Cardiff and Vale Trust, Cardiff, United Kingdom

## Abstract

**Aims:**

To measure the proportion of Attention Deficit Hyperactivity Disorder (ADHD) referrals that result in a positive diagnosis and medication prescription at a community mental health team (CMHT) in Cardiff.

To compare patient journeys from referral to diagnosis – documenting the use of GP mental health liaison, private psychiatrists, questionnaires and CMHT appointments.

To measure the proportion of patients with a pre-existing private diagnosis of ADHD that subsequently received a positive diagnosis by the CMHT.

**Methods:**

230 referrals were made to Pendine CMHT in 2022 for consideration of ADHD. Patient e-records were manually reviewed over a 12-month period following initial referral.

We recorded whether a patient had a pre-existing private diagnosis and whether they were subsequently diagnosed with ADHD by the CMHT. It was also recorded if medication was prescribed or if an alternative diagnosis was suggested.

We recorded whether the patient was asked to see GP mental health liaison team, fulfil an ADHD questionnaire, or attend a doctor appointment before a diagnosis of ADHD was made or refuted.

For positive diagnoses, patient records were reviewed to record whether this diagnosis was later changed on subsequent appointments.

**Results:**

Of 230 referrals, 32 received a CMHT diagnosis of ADHD (14%) and 25 were prescribed medication for ADHD (11%).

Of the 25 patients who received a positive diagnosis and medication, 4 had the diagnosis changed on a subsequent appointment and medication stopped.

21 patients had a pre-existing private sector diagnosis of ADHD, of which 9 (43%) were given a positive diagnosis by CMHT and 8 (38%) were prescribed medication.

Of 230 total referrals, 33 were asked to see their GP mental health liaison team for information gathering before re-referral to the CMHT. 112 were asked to complete a questionnaire before an appointment would be considered. 87 were given a consultant psychiatrist appointment at CMHT.

When ADHD was not diagnosed, the most common alternative diagnoses suggested by the CMHT were anxiety, substance misuse or emotional dysregulation (36, 23 and 9 patients respectively).

**Conclusion:**

Referrals to the CMHT for ADHD assessment result in a low rate of positive diagnosis and even lower rates of medication prescription, even for those with an existing private diagnosis.

Patient journeys vary markedly, which we propose reflects the variable quality of referrals and pressure on the CMHT to protect clinic time.

Future work to create ADHD referral guidance is needed to ensure better patient experience and proper utilisation of secondary mental health resource.

